# Supplementary figures and images for: Species-specific identification of donkey-hide gelatin and its adulterants using marker peptides
Source: PLoS One. 2022 Aug 12;17(8):e0273021. doi: 10.1371/journal.pone.0273021 (PMC9374224; doi:10.1371/journal.pone.0273021)

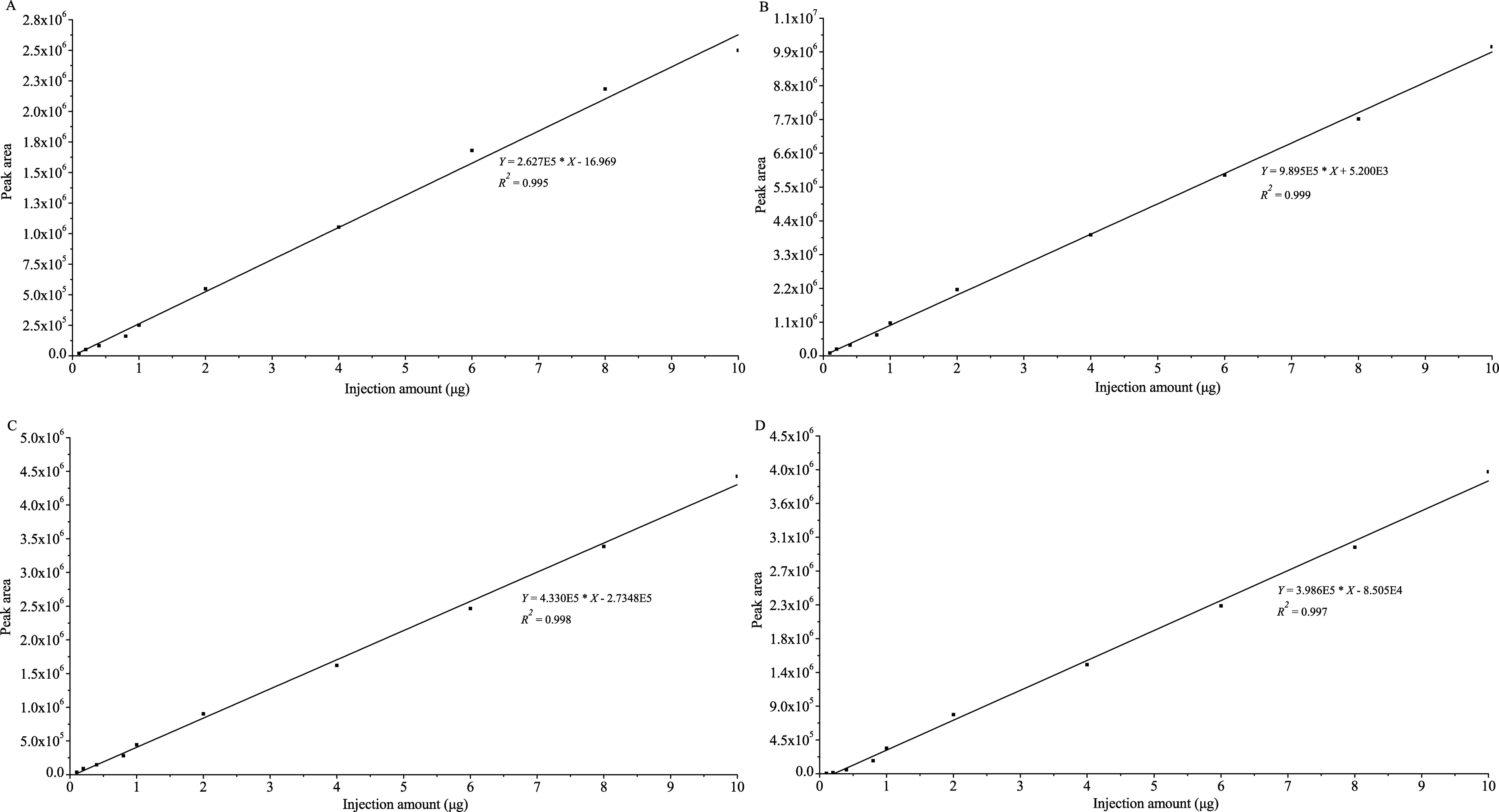

Supplement: S1 Fig — Linearity of the LC–MS/MS MRM method for the analysis of the four marker peptides: (A) 570.4→698.3 for LA1, (B) 386.2→402.2 for MA1, (C) 641.3→726.4 for NA1, and (D) 590.8→894.5 for ZA1. (TIF) [file pone.0273021.s001.tif]

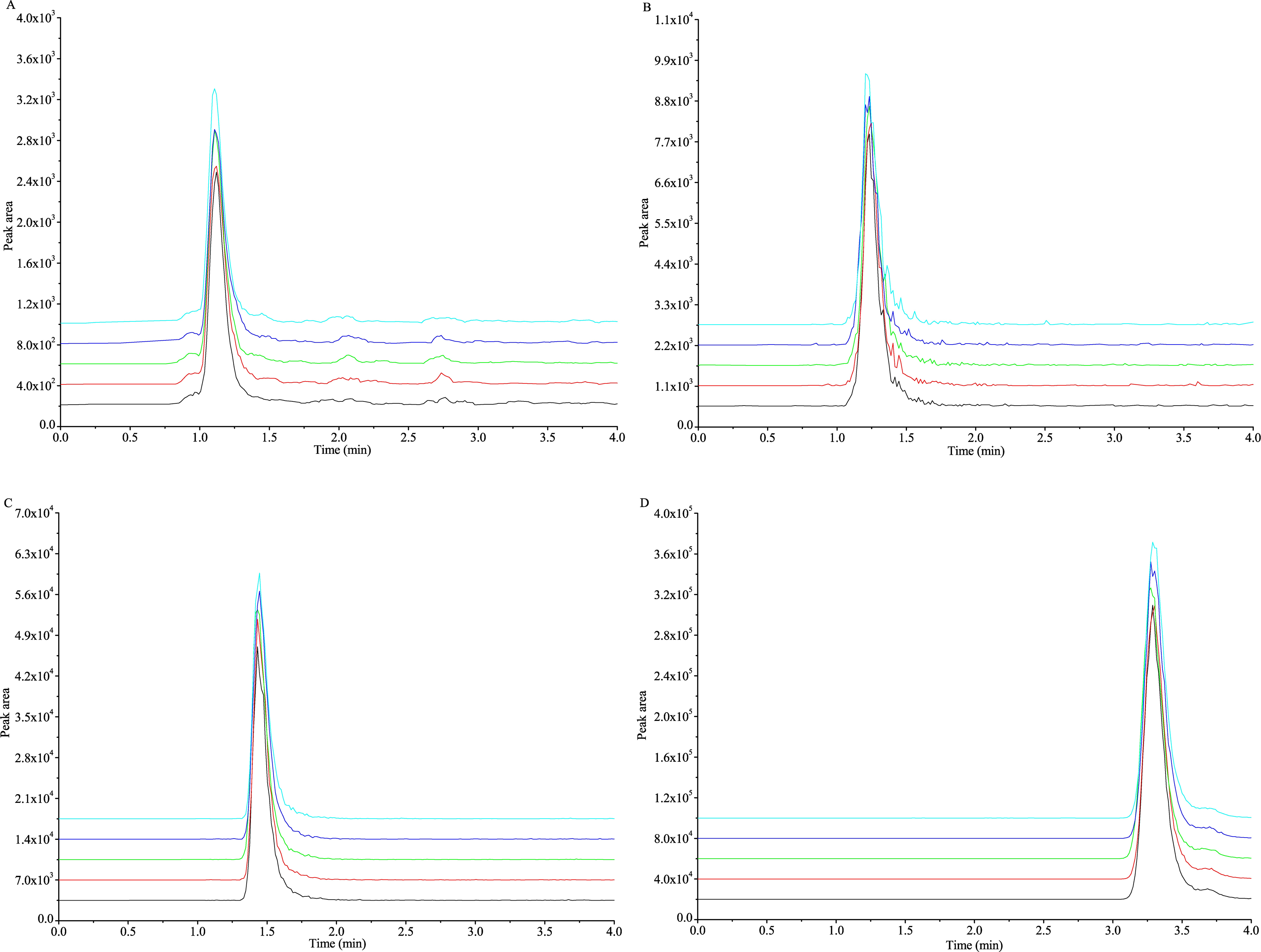

Supplement: S2 Fig — (A) Donkey-hide gelatin, (B) horse-hide gelatin, (C) cattle-hide gelatin, and (D) pig-hide gelatin. Five replicates for each gelatin sample were measured independently, and the results were reproducible. (TIF) [file pone.0273021.s002.tif]

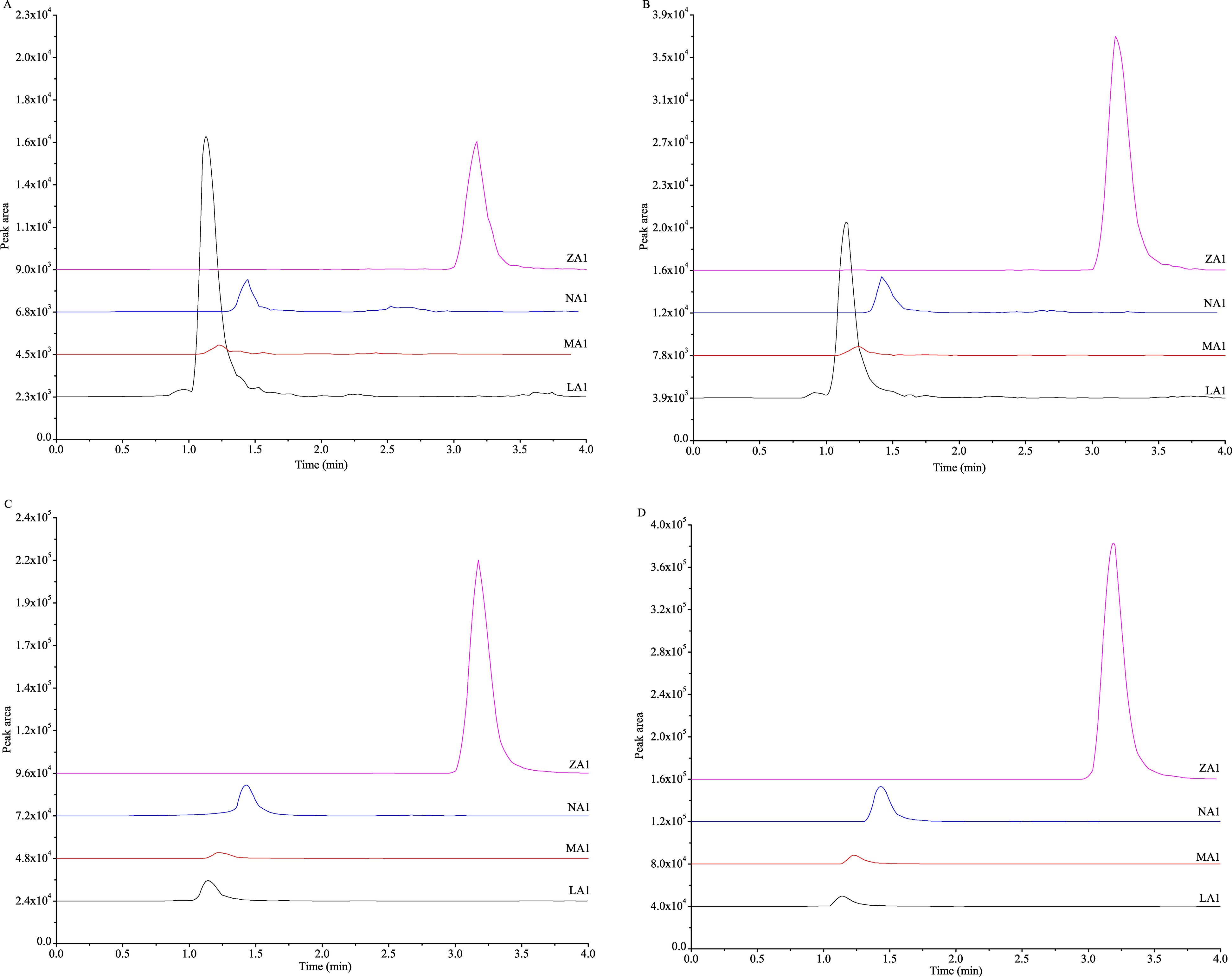

Supplement: S3 Fig — Donkey-hide gelatin samples were mixed with the following amounts of horse-hide gelatin, cattle-hide gelatin and pig-hide gelatin: (A) 0.1%, (B) 0.5%, (C) 5.0%, and (D) 10.0%. The addition of two of the three other gelatins could be detected at a level of 0.1% of the total weight. (TIF) [file pone.0273021.s003.tif]

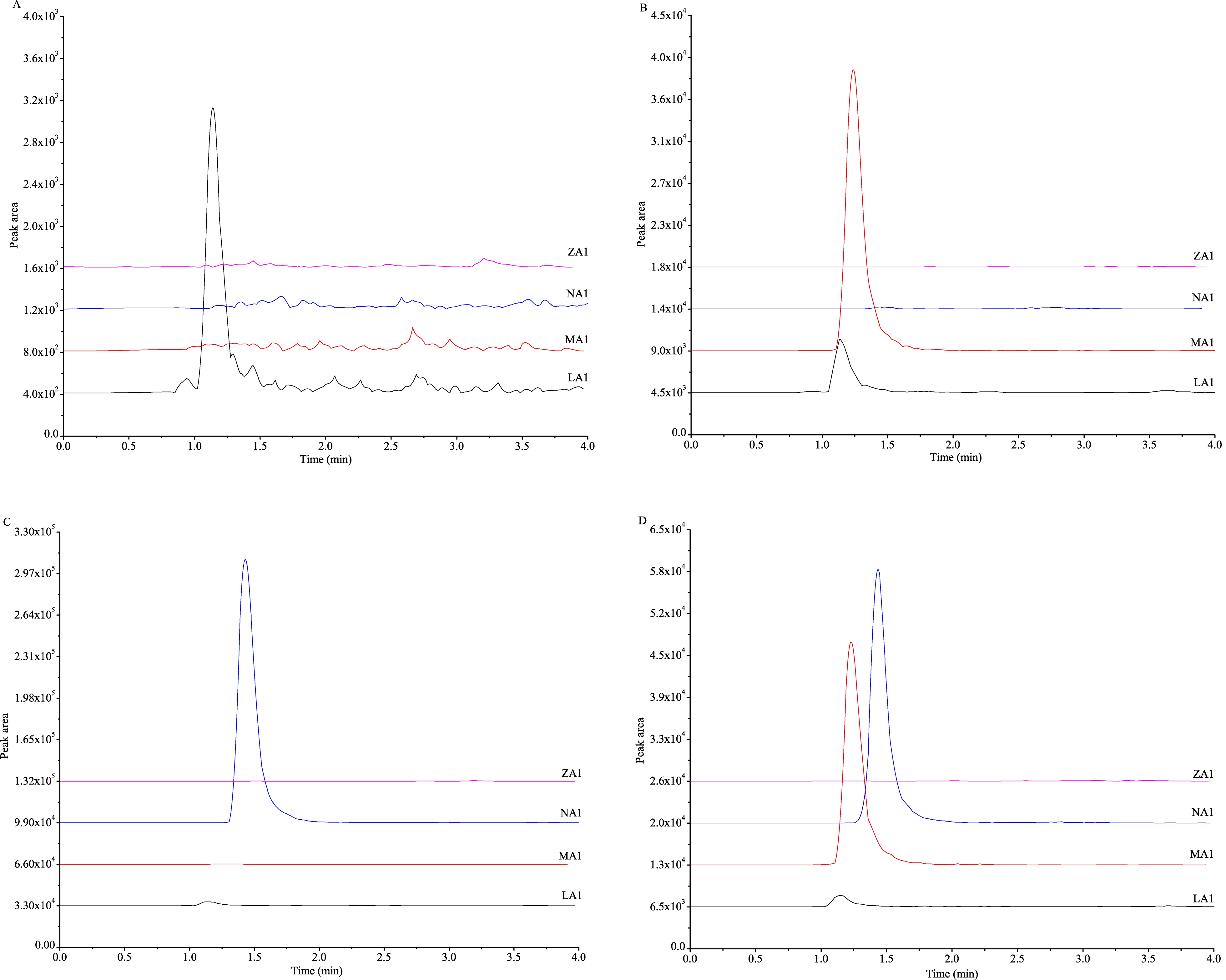

Supplement: S4 Fig — (A) Identified as an authentic donkey-hide gelatin product. Samples (B), (C) and (D) were identified as counterfeit commercial donkey-hide gelatin products adulterated with horse-hide gelatin and cattle-hide gelatin. (TIF) [file pone.0273021.s004.tif]
