# Supplementary material for: Species-specific identification of donkey-hide gelatin and its adulterants using marker peptides
Source: PLoS One. 2022 Aug 12;17(8):e0273021. doi: 10.1371/journal.pone.0273021 (PMC9374224; doi:10.1371/journal.pone.0273021)
Supplement: Attachment — Submitted filename: Response to Reviewers.docx [file pone.0273021.s010.docx]

Dear Dr/ Prof. Chun-Hua Wang,

Thank you for providing us this opportunity to further revise our manuscript entitled “Species-specific identification of donkey-hide gelatin and its adulterants using marker peptides” (ID: PONE-D-22-11303R1).

We appreciate the very positive and constructive comments from the reviewers. Those comments are all valuable and very helpful for revising and improving our manuscript. We have carefully revised the article according to the comments of reviewers, and modifications are marked in red in this revised manuscript. Moreover, in order to facilitate the review process, we provided a point-by-point response to each of the comments. The precise page and line in the revised manuscript where each change was made in response to the comments were provided as well.

We believe that we had addressed all the concerns/questions raised by the reviewers and hope that

the manuscript is now acceptable to PLOS ONE. Should you have any additional questions, please do not hesitate to contact us.

We looking forward to hearing from you.

Yours sincerely,

Jin-ju Zhang

Corresponding author: Ying Zhang
College of pharmacy, Jinan University
E-mail: 1549635053@qq.com; carolynzy@163.com

**Responses to comments about Journal Requirements**

Comment #1: Please review your reference list to ensure that it is complete and correct…and full reference for the retraction notice.

Response #1: We have carefully reviewed the references list to ensure that the references meet PLOS ONE's requirements.

**Responds to comments from the reviewers**

**Reviewer #3:**

Comment #1: If this LC-MS/MS of marker peptide is not the first investigation on DHG and its adulterants (The study presented in this thesis is a comprehensive one of the first investigations to focus specifically on the identification of gelatins using marker peptides.), what is the innovation of this LC-MS/MS? Please compare the proposed method and the published LC-MS/MS method of marker peptide on the identification of DHG.

Response **#**1: Thanks for your good comments. As you mentioned before, previous studies have focus on the identification of donkey-hide gelatin based on the marker peptides. So, we have deleted the statement of “one of the first investigation…”. LC-MS/MS based on proteomics experiments are commonly used on the discovery of marker peptides, meanwhile the reference protein database used to predict the theoretical marker peptides and specified search is essential for identifying peptides. After the discovery of marker peptides, it is also a challenge to build a fast, sensitive and accurate method based on the marker peptides. In our study, to obtain enough candidate marker peptides, we used both the α1 (I) and α2 (I) chains to predict the theoretical marker peptides. Although we focused on the marker peptides derived from type I collagen, we used all the reference proteins of four animals from UniProt as searched database. Our results might provide a reference to discover other marker peptides which derived from non-collagen proteins such as keratins. Using the marker peptides, we build an MRM method which allows a fast identification of four kinds of gelatins in 15 minutes with good specialty and sensitivity. We also collected and tested a variety kinds of commercial donkey-hide gelatin products which covers large brands, and the results might help us understand the real situation of the donkey-hide gelatin on the market.

Comment #2: Please add the method of identification of DHG of Chinsed Pharmacopoeia 2020 version and discuss the limitations of the CHP2020 method briefly and show the proposed LC-MS/MS can be helpful to improve the CHP 2020 method in Introducation part but not in the discussion part.

Response #2: This is an interesting question. In Chinese Pharmacopoeia 2020 edition, quantitative determination of A1 and A2 peptides derived from donkey skins are used to assess the quality of donkey-hide gelatin. The total amount of A1 and A2 should reach at least 0.15%. The donkey-hide gelatin with a low level of A1 and A2 may be considered as products with an insufficient amount of donkey skin. However, marker peptides of A1 and A2 can be synthesized and added to donkey-hide gelatin products to satisfy the detection criterion. Therefore, donkey-hide gelatin products with an insufficient amount of donkey skin cannot be completely avoided only by quantitative analysis of the amounts of marker peptides. Unfortunately, not only our current research but also other studies cannot provide a good solution to avoid the artificial addition of the marker peptides. We've been paying attention and trying to solve this problem in the future. Alternatively, it may be more appropriate to discuss this issue in the “Discussion” section. We have considered whether proteins which are difficult to synthesize could be used to identify different gelatins. The Parallel Reaction Monitoring (PRM) which is commonly used to quantify proteins may be used to analysis the collagens or other proteins in different gelatins. If we establish a PRM method which can specifically identify the donkey-hide gelatin based on the quantitative determination of protein components, the problem may be solved satisfactorily at last.

Comment #3: Line113-115, … Chinese Pharmacopoeia 114 and the quality of every gelatin sample complied with Chinese Pharmacopeia 115 requirements. Please add the version of the Chinese Pharmacopoeia. for example,2020 version.

Response #3: We have added the version of Chinese Pharmacopoeia in the manuscript (Page 6, Line 112-113).
